# Probiotic modulation of gut microbiota by *Bacillus coagulans* MTCC 5856 in healthy subjects: A randomized, double-blind, placebo-control study

**DOI:** 10.1097/MD.0000000000033751

**Published:** 2023-05-17

**Authors:** Muhammed Majeed, Kalyanam Nagabhushanam, Lakshmi Mundkur, Shaji Paulose, Hema Divakar, Sudha Rao, Sivakumar Arumugam

**Affiliations:** a Sami-Sabinsa Group Limited, Bangalore, Karnataka, India; b Sabinsa Corporation, East Windsor, NJ; c Divakars Speciality Hospital, Bangalore, Karnataka, India; d Genotypic Technology Private Limited, Bangalore, Karnataka, India.

**Keywords:** *Bacillus coagulans* MTCC 5856, bacterial persistence, gut microbiome, metagenome analysis, next-generation sequencing, quantitative reverse transcription PCR

## Abstract

Probiotics are known to rebalance the gut microbiota in dysbiotic individuals, but their impact on the gut microbiome of healthy individuals is seldom studied. The current study is designed to assess the impact and safety of *Bacillus coagulans (Weizmannia coagulans*) microbial type culture collection 5856 (LactoSpore®) supplementation on microbiota composition in healthy Indian adults.

**Methods:**

The study participants (N = 30) received either LactoSpore (2 billion colony-forming units/capsule) or placebo for 28 days. The general and digestive health were assessed through questionnaires and safety by monitoring adverse events. Taxonomic profiling of the fecal samples was carried out by 16S rRNA amplicon sequencing using the Illumina MiSeq platform. The bacterial persistence was enumerated by quantitative reverse transcription-polymerase chain reaction.

**Results:**

Gut health, general health, and blood biochemical parameters remained normal in all the participants. No adverse events were reported during the study. Metataxonomic analysis revealed minimal changes to the gut microbiome of otherwise healthy subjects and balance of *Bacteroidetes* and *Firmicutes* was maintained by LactoSpore. The relative abundance of beneficial bacteria like *Prevotella, Faecalibacterium, Blautia, Megasphaera*, and *Ruminococcus* showed an increase in probiotic-supplemented individuals. The quantitative polymerase chain reaction analysis revealed highly variable numbers of *B. coagulans* in feces before and after the study.

**Conclusion:**

The present study results suggest that LactoSpore is safe for consumption and does not alter the gut microbiome of healthy individuals. Minor changes in a few bacterial species may have a beneficial outcome in healthy individuals. The results reiterate the safety of *B. coagulans* microbial type culture collection 5856 as a dietary supplement and provide a rationale to explore its effect on gut microbiome composition in individuals with dysbiosis.

## 1. Introduction

The Food and Agricultural Organization of the United Nations and the World Health Organization defines probiotics as live microorganisms that, when administered in adequate amounts, confer a health benefit on the host.^[1]^ *Bifidobacteria, Lactobacilli, Enterococci, Bacillus, Escherichia, Propionibacterium*, and *Saccharomyces* are some of the promising probiotic bacteria^[2]^ which have a beneficial effect on an extensive array of human diseases, including, irritable bowel syndrome (IBS), metabolic syndrome, allergic diseases as well as neurodegenerative diseases.^[3,4]^ Several studies have reported the positive effect of probiotics on restoring the gut microbiome in conditions of gut dysbiosis.^[5]^ However, the impact of probiotics on gut microbiome composition in healthy individuals is not very well established, mainly due to the lack of consensus on the definition of a normal fecal microbial composition.^[6]^ The microbial composition of healthy individuals varies significantly according to age, lifestyle, ethnicity, genetics, and environment.^[7,8]^ In a meta-analysis of probiotic supplementation in healthy adults, Kristensen et al^[9]^ observed a lack of evidence for any effect of probiotics on fecal microbiota composition. In the absence of dysbiosis, the gut microbiome is not expected to change due to the resilience of gut homeostasis.^[10]^ Thus understanding the impact of probiotics on gut microbiome composition in individuals without dysbiosis would provide insight into microbial interactions in vivo and may be an essential feature of the safety of the probiotics in healthy individuals.

*B. coagulans* microbial type culture collection (MTCC) 5856 (LactoSpore^®^, Sami-Sabinsa Group Limited) is a patented gram-positive, endospore-forming, nonpathogenic bacterial probiotic strain. It is a facultative anaerobe that grows optimally at a slightly acidic pH range of 5.5 to 6.2 and a temperature of 37°C.^[11]^ When ingested, the strain produces L(+) lactic acid as a primary product after germination and prevents the growth of pathogenic microbes in the GI tract.^[12]^ The spores of *B. coagulans* MTCC 5856 strain are thermostable, genetically, and phenotypically consistent over years of commercial production and can be processed in various functional food and beverages.^[13–15]^ *B. coagulans* MTCC 5856 has been clinically evaluated for benefits in diarrhea-predominant IBS and improvement of depression in IBS patients^[16,17]^ and was found to be safe and well tolerated at a dose of 2 × 10^9^ colony forming units/day for 30 days.^[18]^ It showed cholesterol-lowering activity in vitro^[15]^ and was evaluated as a synbiotic in combination with prebiotic fibers such as fenugreek galactomannan^[19]^ and cranberry fiber.^[20]^

Several strains of *B. coagulans* are commercially available as probiotic supplements.^[21]^ Moreover, the efficacy of probiotics is reported to be both strain and disease-specific.^[19,20]^ The effect of *B. coagulans* MTCC 5856 on the composition of the gut microbiome has not been evaluated so far. In the current study, we explored the impact of *B. coagulans* MTCC 5856 supplementation on the changes in the composition and abundance of gut microbiota by 16S rRNA amplicon next-generation sequencing using the (Illumina, USA) platform. Further, the impact of *B. coagulans* MTCC 5856 consumption on vital signs and other safety parameters were evaluated in healthy individuals.

## 2. Material and methods

### 2.1. Test product description

Each active capsule contained *B. coagulans* MTCC 5856, 2 billion colony-forming units/capsule formulated in maltodextrin, and the placebo capsules contained only maltodextrin. The viable count of *B. coagulans* MTCC 5856 was determined as per the method described earlier.^[14]^

### 2.2. Study participants

Thirty male and female adult healthy volunteers aged between 25 and 55 years with a body mass index between 20 and 27 kg/m^2^ (both inclusive), mixed diet consuming nonsmokers, willing to come for regular follow-up visits and avoid any prebiotic and probiotic food supplements, laxatives, and foods having laxative effects were recruited. The exclusion criteria included antibiotic use, any underlying gastrointestinal complaints, presence of inflammatory disorders and mental illness, a history of drug or alcohol abuse, participation in a clinical study in the last 90 days, and not willing to abide by the study procedures or not willing to provide stool samples. The study was conducted according to the ICH-GCP E6 R2 guidelines, Declaration of Helsinki (2013), after getting approval from the Institutional Ethics Committee (Hairline Research Ethics Committee, Bangalore, India). The study protocol with number CPL/65/LS/May/18 was approved on September 17, 2018, by the Independent Ethics Committee at Divakar’s Speciality Hospital prior to the initiation of the study at the site. All the participants signed written informed consent, and the study was registered at Clinical Trial Registry India (www.ctri.nic.in CTRI/2018/10/015913).

### 2.3. Randomization and masking

The study was conducted as a randomized, double-blind, placebo-controlled trial at Divakar Hospital, Bangalore, India. The subjects (N = 30) were randomized, using a random number generator. An alphanumeric code was generated for both the investigational products to improve the blindness of the study and the concealment of allocations. Block randomization (only 1 block) was followed wherein the subjects were randomized to receive either of the 2 investigational products. The study supplements were coded and supplied to the study site, in a manner to maintain the blind throughout the study. The randomization codes were kept strictly confidential and were accessible only to authorized persons on an emergency basis as per the Sponsor's standard operating procedures until the time of unblinding.

### 2.4. Study design

**Figure 1.**
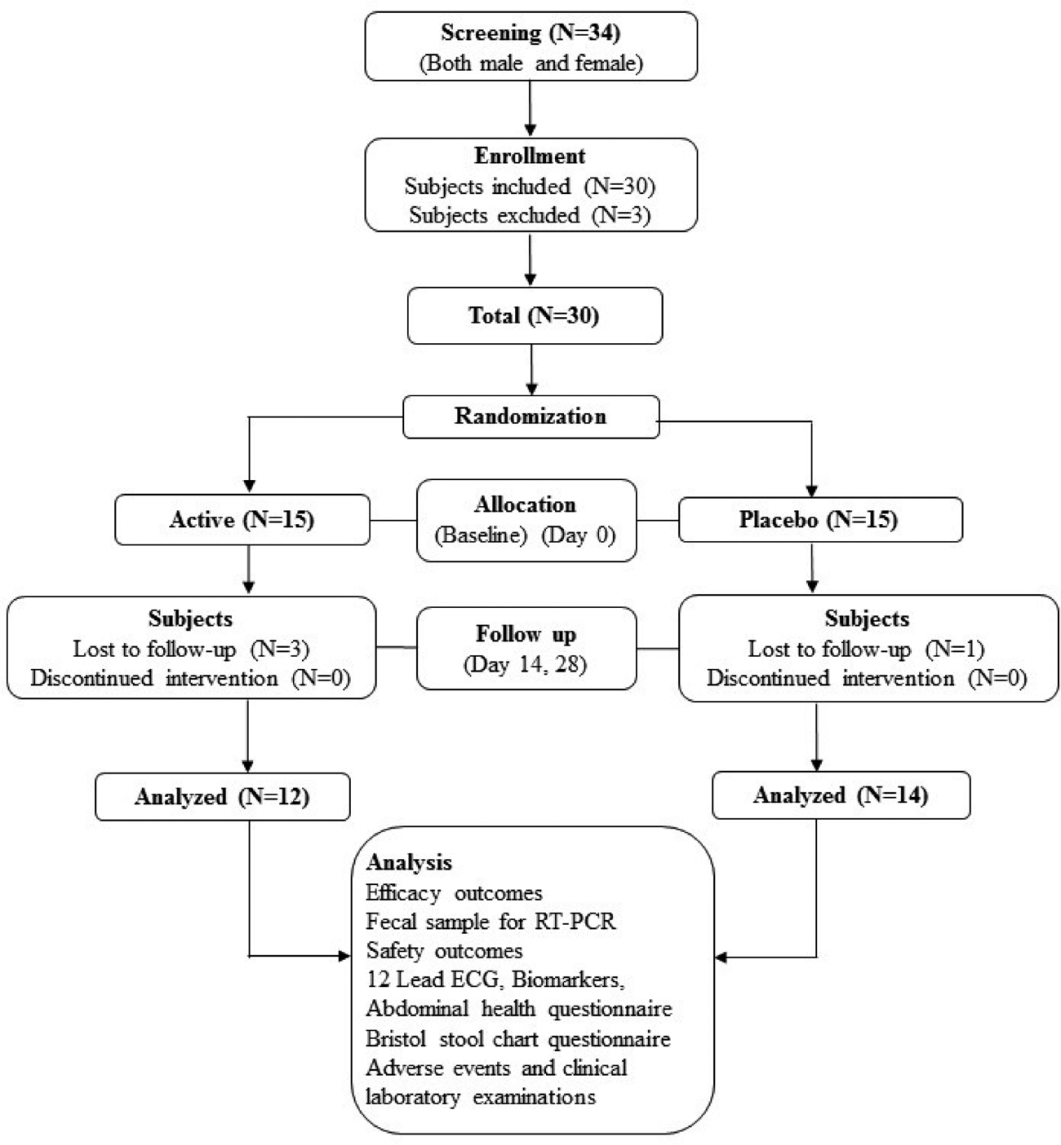
Consort diagram – flowchart of study procedures, from eligible 30 subjects who fit into inclusion and exclusion criteria, 26 subjects completed the study. At every follow-up visit, study evaluations were made in both study groups.

Statistically, a significant sample size was considered for this study. A total of 30 subjects including drop-outs were considered for this clinical study. Further, all the subjects were randomly allocated into 2 treatment groups Active and Placebo. Subjects were randomly assigned to receive either 1 capsule of *B. coagulans* MTCC 5856 or placebo orally once daily after dinner (Fig. 1). The study participants had 4 visits to the hospital, screening or visit 1 (−3 days), baseline visit 2 (day 0), visit 3 (day 14), and final visit 4 (day 28). Further details are available in Supplementary Methods, Supplemental Digital Content, http://links.lww.com/MD/I984.

### 2.5. Study objective

The objective of the study was to assess the impact of *B. coagulans* MTCC 5856 on the fecal microflora and its safety in healthy individuals. The assessments include, 16S Illumina-based sequencing of fecal samples on day 0 and day 28, reverse transcription-polymerase chain reaction (RT-PCR)-based enumeration of *B. coagulans.* The safety parameters included Abdominal Health Questionnaire, Bristol Stool Chart, routine hematological and biochemical evaluations, 12-lead electrocardiogram and serum biomarkers, high-sensitivity C-reactive protein (hs-CRP), interleukin (IL)-10, and tumor necrosis factor (TNF)-α.

### 2.6. DNA extraction and sequencing

For microbiome analysis, fecal samples were collected at baseline before dosing and final visit in fecal collection Tube DNA/RNA Shield™ (Zymo Research, USA) and stored at −80°C until use. The fecal sample (approximately 500 μL) was lysed with lysozyme (Sigma # L6876), followed by Proteinase K and RNase treatment to remove proteins and RNA. The genomic DNA was extracted from each frozen fecal sample using the Qiagen DNeasy column (Qiagen, USA) (#69506) and was quantified using Nanodrop2000 (Thermo Fisher Scientific, USA).

### 2.7. Library preparation and sequencing

Libraries were constructed using 50 ng of genomic DNA by a 2-step PCR-based workflow. In round 1 PCR, the 16S rRNA gene V3 to V4 regions were first amplified for 26 cycles using region-specific proprietary primers developed at Genotypic Technology Pvt. Ltd., Bangalore, India, using KAPA HiFi Hot Start PCR Kit (KAPA Biosystems Inc., Boston, MA). The PCR amplicons were further amplified for 10 cycles to add Illumina sequencing barcoded adaptors (Nextera XT v2 Index Kit, Illumina, CA). The libraries were normalized and pooled for multiplex sequencing. The normalized sample was denatured for 5 minutes using 0.2 N NaOH and neutralized by HT1 Buffer (Illumina). Denatured libraries were further diluted to the concentration of 13 pM and loaded into an Illumina MiSeq v3 600 cycles cartridge (Illumina) to read 275 bp length for forward (Read 1) and reverse (Read 2) reads in paired-end mode.

After the completion of the sequencing, the data were demultiplexed using bcl2fastq software v 2.20 (Illumina, USA),^[22]^ and Fast Q files were generated based on the unique dual barcode sequences. The sequencing quality was assessed using the Fast QC v 0.11.8 software (Babraham Bioinformatics, USA).^[23]^ The adapter sequences were trimmed and bases above Q30 were considered for analysis. Low-quality bases were filtered off during reading pre-processing and used for downstream analysis. The supplementary document provides the details of the sequencing.

### 2.8. Metagenome analysis

From the Illumina paired-end raw reads of 52 samples, the reads having a V3 to V4 primer sequence and high-quality bases were filtered. Short overlapping forward and reverse reads from the same fragment were joined using Fastq-join^[23]^ to form sequences of the V3 to V4 hypervariable 16S rRNA region. These stitched reads were considered for microbiome search using the Quantitative Insights Into Microbial Ecology (QIIME)^[24]^ pipeline. The query sequences were clustered using the UCLUST method^[25]^ against a curated chimera-free 16S rRNA database (GreenGenes v13.8).^[26]^ The taxonomies were assigned using the RDP classifier^[27]^ to these clusters at ≥97% sequence similarity against the reference database, which resulted in the generation of a biom file that was used for advanced analysis and visualization. The biom file contains information about the number of reads assigned to taxa. The details, such as reads utilized to identify the microbiome and the number of operational taxonomic units (OTUs) picked for each sample, were identified using QIIME scripts. Relative abundance from phylum to species was calculated from the read counts assigned to OTU, divided by the total utilized reads for microbiome search. The R package for nonnegative matrix factorization was used to generate boxplots.^[28]^

### 2.9. Comparative analysis across a group of samples

The difference in relative abundance was compared within the group and between the active and placebo group based on the data from baseline and final visit samples. The focus of the comparative analysis was to quantify some of the bacterial species that predominate the human intestine (*Clostridium coccoides* group, *Bacteroides fragilis, Bifidobacterium, Atopobium cluster, Eubacterium rectale, Clostridium histolyticum* subgroup, and *Prevotella*), 8 potential pathogens (*Clostridium difficile, Clostridium perfringens, Enterobacteriaceae, Enterococcus* spp., *Streptococcus* spp., *Staphylococcus* spp., *Escherichia coli*, and *Pseudomonas* spp.) and a few Lactobacilli groups were also quantified in the active and placebo groups.

### 2.10. Bioinformatics analysis

Alpha diversity was calculated using the Shannon, Simpson, Chao1, and OTU diversity matrices. Beta diversity was determined by principal coordinate analysis (PcoA) using unweighted and weighted UniFrac metrics. Emperor 3D viewer was used to visualize the plots. Taxon differential abundance across the groups was performed in QIIME (QIIME: group significance.py) to examine whether observation counts (i.e., OTUs and Microbial taxon) are significantly different between the groups at baseline and final visit. Before the final community quality control, the OTU table was collapsed at each taxonomic level (i.e., Phylum–nus; QIIME: collapse_taxonomy.py), with counts representing the relative abundance of each microbial taxon. Differences in the mean abundance of taxa between sample groups were calculated using Kruskal–Wallis nonparametric statistical tests. The taxon was ranked with *P* values of most to least significant (*P* < .05) provided alongside false discovery rate, and Bonferroni corrected *P* values, and then the taxon was ranked from most to least significant (*P* < .05).

### 2.11. RNA extraction for the rRNA-targeted RT-qPCR

The RNA was extracted from the fecal samples by the Trizol method, using the RNAeasy kit (Qiagen, USA) and NucleoSpin RNA stool kit (Takara Bio Inc.) as per manufacturer guidelines. The extracted RNA was quantified, and quality was assessed by Tape station (Agilent Technologies Inc., USA) and Bioanalyzer (Agilent Technologies Inc., USA).

### 2.12. 16S rRNA-specific primer design

By using 16S rRNA sequences obtained from the National Center for Biotechnology Information database for the *Bacillaceae* family, multiple alignments of the target groups and reference *B. coagulans* 16S rRNA was constructed using the Clustal W program (Thompson et al, 1994; PMID: 7984417). After comparing the sequences, potential primer target sites were identified for specific detection of *B. coagulans*. The designed primer specificity was assessed by performing primer BLAST against the non-redundant database by submitting the sequences to the National Center for Biotechnology Information Primer-BLAST program.

### 2.13. Primers used

Primer sequence used for a 1-step reaction for complementary DNA (cDNA) conversion –AGCCGCCTGCGCGCGCTTTACGCCC

Primer sequences used for cDNA amplification

Forward primer – AGTGCCGTTCGAACAGGGCGGCGCC

Reverse primer – AGCCGCCTGCGCGCGCTTTACGCCC

### 2.14. Establishment of an analytical system for the human fecal microbiota by qRT-PCR

RT-qPCR was conducted in a 1-step reaction using the SuperScript IV 1-Step RT-PCR method. The methodology involves the use of *B. coagulans*-specific reverse primer of 16S rRNA gene for the cDNA conversion and its direct detection by standard SYBR green chemistry. Briefly, 10 ng of the extracted RNA and 2 mM 16S rRNA reverse primer were incubated at 65°C for 5 minutes and mixed with the reverse transcriptase mix for 15 minutes. The qPCR reactions were performed using the Agilent Stratagene 3005 system., using 2 µL of the cDNA and the Agilent Brilliant SYBR (Agilent Technologies Inc., USA) green dye in an Agilent Strata gene 3005 system. For standardizing the protocol, total RNA fractions corresponding to 10^5^ cultured cells (spores and vegetative cells) were enumerated by RT-qPCR. Serial dilutions of the samples were analyzed to check the efficiency of primer, using the standard curve, and the same was used against all test samples to estimate the number of *B. coagulans* cells. The amplified signal was judged as positive when it was more than that of 10^1^ standard cells, and the cell count between 10^−1^ and 10^4^ against the standard curve was considered for the enumeration of *B. coagulans*. The final cell count was estimated by the formula.

Total cells = number of cells determined by standard curve* (total yield/total weight of fecal matter used for extraction).

The procedure is detailed in Supplementary Methods, Supplemental Digital Content, http://links.lww.com/MD/I984.

### 2.15. Safety analysis

The participants underwent various biochemical and hematological tests before and after the intervention. Vital signs were monitored at all 4 visits, including blood pressure, respiratory rate, pulse rate, and physical examination. The routine laboratory parameters of safety, that is, hematology, lipid profile, serum biochemistry, human immunodeficiency virus, hepatitis B-virus, and hepatitis C-virus, 12-lead electrocardiogram, were measured using standard laboratory techniques at screening and final visits. The levels of hs-CRP, liver enzymes, and biomarkers (IL-10, and TNF-α) were measured at baseline and final visits by routine methods to ensure the safety of supplementation.

### 2.16. Gut health

Subjects completed the Abdominal Health Questionnaire during the intervention period on days 0, 14, and 28. This questionnaire was used to collect information on the presence and severity of gut health, ranked from none to severe. The consistency of stools was recorded using the Bristol Stool Scale, which ranges from 1 to 7 and a high score indicates looser stool. Adverse effects, if any, were recorded at each study visit.

### 2.17. Statistical analysis of clinical data

All subjects in the study with relevant safety data were considered for the analysis.

A descriptive analysis of demographic characteristics, vital signs, laboratory parameters, and biomarkers was performed. The normality of the data was checked by Shapiro–Wilk test. *P* value was derived using a chi-square test for categorical parameters and paired *t* test or Wilcoxon signed rank test for numeric parameters based on normal distribution of the data for intergroup comparisons. The Mann–Whitney test was used to compare the placebo with probiotic-treated group. Continuous measurements were presented as mean ± SD and results on categorical measurements were presented in percentage. Adverse events are presented in frequency and percentage (%). Clinical laboratory outcomes were assessed descriptively. Mean, standard deviation, minimum and maximum were derived from the data. The metagenomic data was analyzed by Kruskal–Wallis nonparametric statistical tests as described in the Bioinformatic analysis section.

Statistical Analysis Software of version 9.4 was used for analysis, and *P* < .05 was considered statistically significant.

## 3. Results

A total of 34 subjects were screened for the study and 30 of them who met the eligibility criteria were enrolled in the study. Subjects were randomly assigned to either the placebo (n = 15) control group (n = 15). The treatment adherence was 99 % in participants who completed the study. Twelve subjects completed the study in the active group, while 14 completed the study in the placebo. Four subjects withdrew from the study and their data was not considered for the statistical analysis. The demographic characteristics of the 2 groups were comparable at baseline. The mean age of the subjects was 37.67 ± 10.65 in the active group, while it was 39.50 ± 9.15 in the placebo (Table 1). Details of recruitment, randomization, and study flow are shown in CONSORT diagram (Fig. 1).

**Table 1:** Demographic characteristics of subjects selected for the trial.

Parameters	Active		Placebo	
	Baseline	Final visit	Baseline	Final visit
N (number of subjects)	15	12	15	14
Age in years, mean (SD)	37.67 (10.65)	–	39.50 (9.15)	–
Female	9	7	10	9
Male	6	5	5	5
Height (mt)	1.67 (0.06)	1.67 (0.06)	1.64 (0.07)	1.64 (0.07)
Body weight in kg, mean (SD)	70.58 (7.28)	71.00 (6.92)	68.50 (8.84)	69.43 (8.90)
BMI, mean (SD)	25.29 (1.29)	25.45 (1.18)	25.44 (1.50)	25.80 (1.52)

Values expressed as mean (SD).

BMI = body mass index, SD = standard deviation.

### 3.1. Impact on microbial diversity

#### 1.3.1. Alpha diversity.

**Figure 2.**
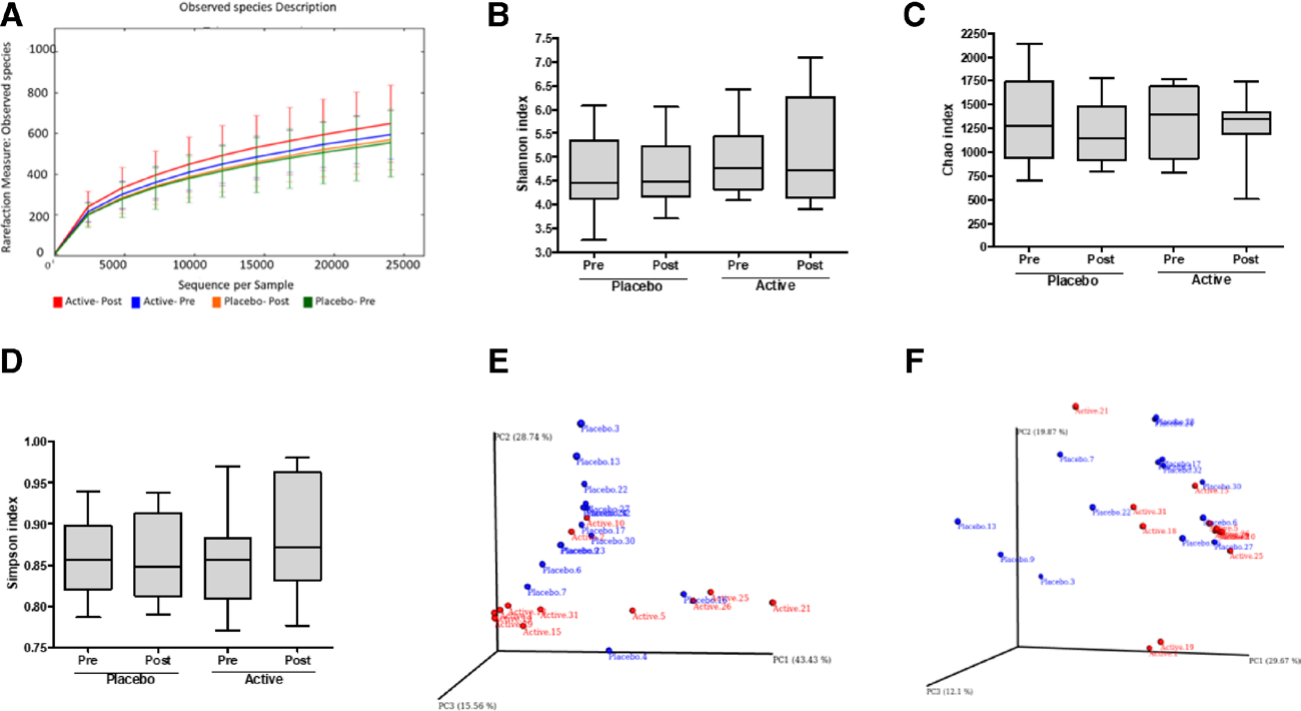
(A) Rarefaction plot for 52 samples of V3 to V4 region at a depth of 24,000. The image was plotted against the number of sequences per sample on the X-axis versus diversity index on Y-axis. The samples are colored by their respective names. As the sequencing depth increased, the number of observed species (OTUs) also increased. Eventually, the curves began to plateau, indicating that as the number of extracted sequences increased, the number of OTUs detected decreased; Box plot representing alpha diversity measured by (B) Shannon, (C) Chao1, (D) Simpson for the Placebo and Active. The line inside the box represents the median, while the whiskers represent the lowest and highest values within the 1.5 interquartile range (IQR); (E and F) The dots represent outliers principal coordinate analysis (PCoA) plots of the active (n = 12), versus placebo group (n = 14) specific taxa with significant (*P* < .05). PCoA plots of (E) weighted and (F) unweighted Unifrac distance matrices. Axis title indicates percentage variation. OTUs = operational taxonomic units.

From the Illumina paired-end raw reads of samples, the reads having a V3 to V4 primer sequence and high-quality bases were filtered. All the samples were individually analyzed for microbiome diversity and species abundance using QIIME analysis. Rarefaction analysis allows comparisons between communities based on the richness of microbial diversity. Alpha rarefaction was performed at a level of 22,000 reads to include all samples, which is used as a measure of the depth of sequencing and the total data. The rarefaction plot in Figure 2A shows the numbers of observed OTUs per sample which indicated that most of the diversity was already achieved and reached a saturated plateau phase. The difference between the groups and that between the baseline and end of the study was not significant.

Alpha diversity indexes are composite indexes reflecting abundance and consistency. We observed a minimum of 375 and a maximum of 1481 species in all the samples. The richness of annotated species in V3 to V4, Shannon, Simpson, and Chao1, indexes are represented in Figures 2B and C. Shannon index reflects the diversity of OTUs in the samples by giving weightage to species richness, while the Simpson index gives evenness a higher weightage. The Chao1 index estimates species richness based on abundance. The alpha diversity was not significantly different between the baseline and end of the study in both active and placebo, except for a slight increase in the alpha diversity in the active samples.

#### 2.3.1. Beta diversity.

A scatter plot based on PCoA showed clustering of certain samples in active and placebo based on unweighted and weighted UniFrac data. The difference between active and placebo was found to be significant (*P* < .05). The weighted PCoA, UniFrac plot exhibited the relative abundance of OTUs among samples, determining the changes in abundant taxa (Fig. 2D), while the unweighted PCoA UniFrac plot (Fig. 2E and F) represented the phylogenetic distance based on the presence/absence of OTUs among samples, thus determining the changes in rare taxa. In the group-wise analysis, the clustering was observed in the unweighted UniFrac method, which indicated the presence/absence of certain taxa only in those active samples.

### 3.2. Microbiome composition in active and placebo

**Figure 3.**
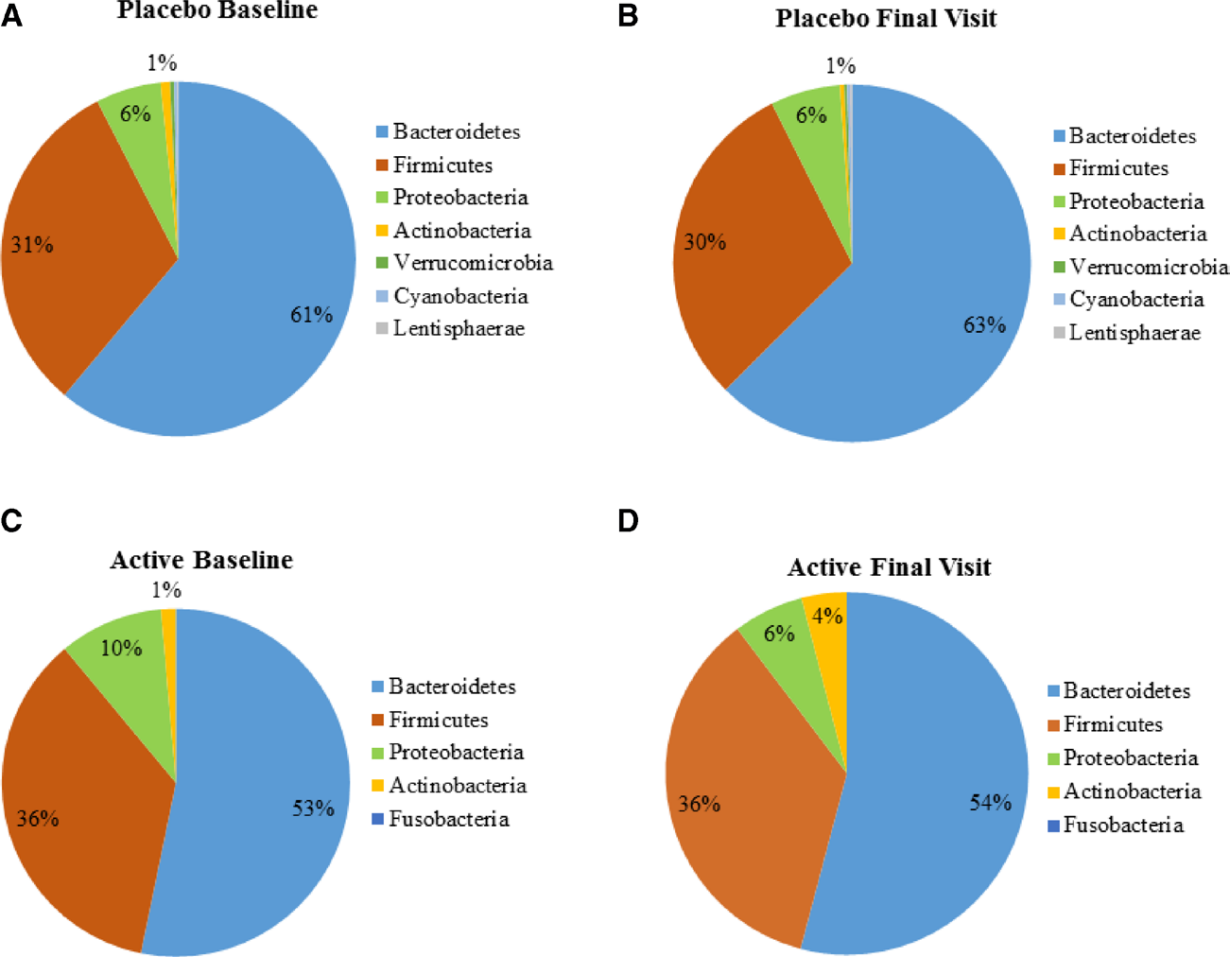
The relative abundance of the bacterial phyla (A) placebo baseline visit; (B) placebo final visit; (C) active baseline and (D) active final visit.

*Bacteroidetes* were the dominant phyla present in all the samples, followed by *Firmicutes, Proteobacteria*, and *Actinobacteria*. The pie chart was generated using matplotlib for the taxa present above the cutoff value of >0.05%. The *Bacteroidetes* contributed to 61% and 63% in placebo and 53% and 54% in probiotic-supplemented individuals at baseline and end of the study respectively. Similarly, no change was observed in the relative abundance of *Firmicutes* (31–30% in placebo and 36–36% in the probiotic group). *Proteobacteria* and *Actinobacteria* remained at 6 and 1% respectively in the placebo while there was a decrease in *Proteobacteria* from 10 to 6% and an increase in *Actinobacteria* from 1 to 4% in the probiotic group. (Fig. 3A–D). The phylum-level bar plots of all the samples are shown in the supplementary section Supplementary Fig. S1, Supplemental Digital Content, http://links.lww.com/MD/I985.

**Figure 4.**
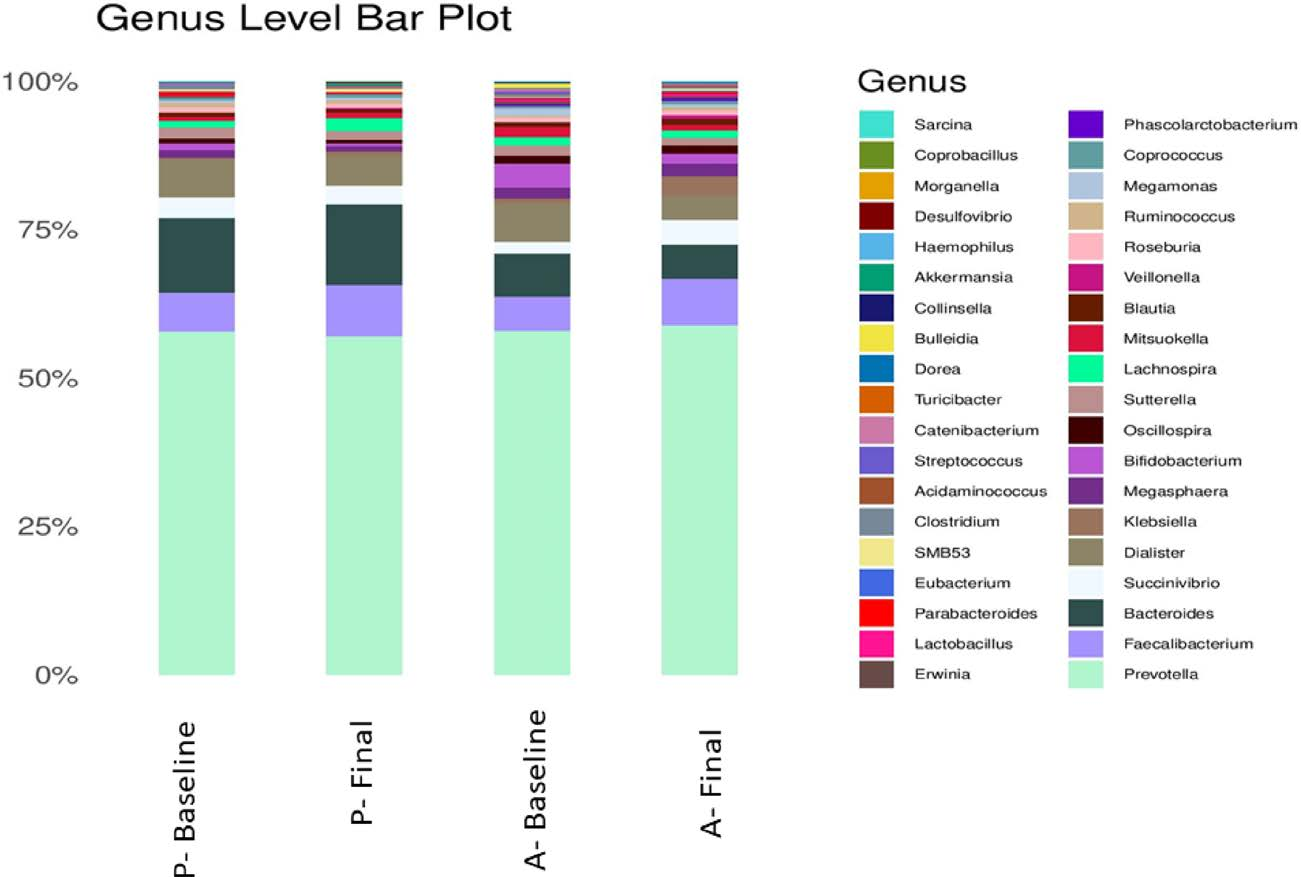
Bar plot showing microbial composition of active and placebo group at baseline and final visit at genus level with its corresponding relative percentages.

At the genus level, *Prevotella* remained high in both placebo and LactoSpore groups, followed by *Faecalibacterium* and *Bacteroides* (Fig. 4). The genus level bar plots of all the samples are shown in the supplementary section Supplementary Fig. S2, Supplemental Digital Content, http://links.lww.com/MD/I986.

**Figure 5.**
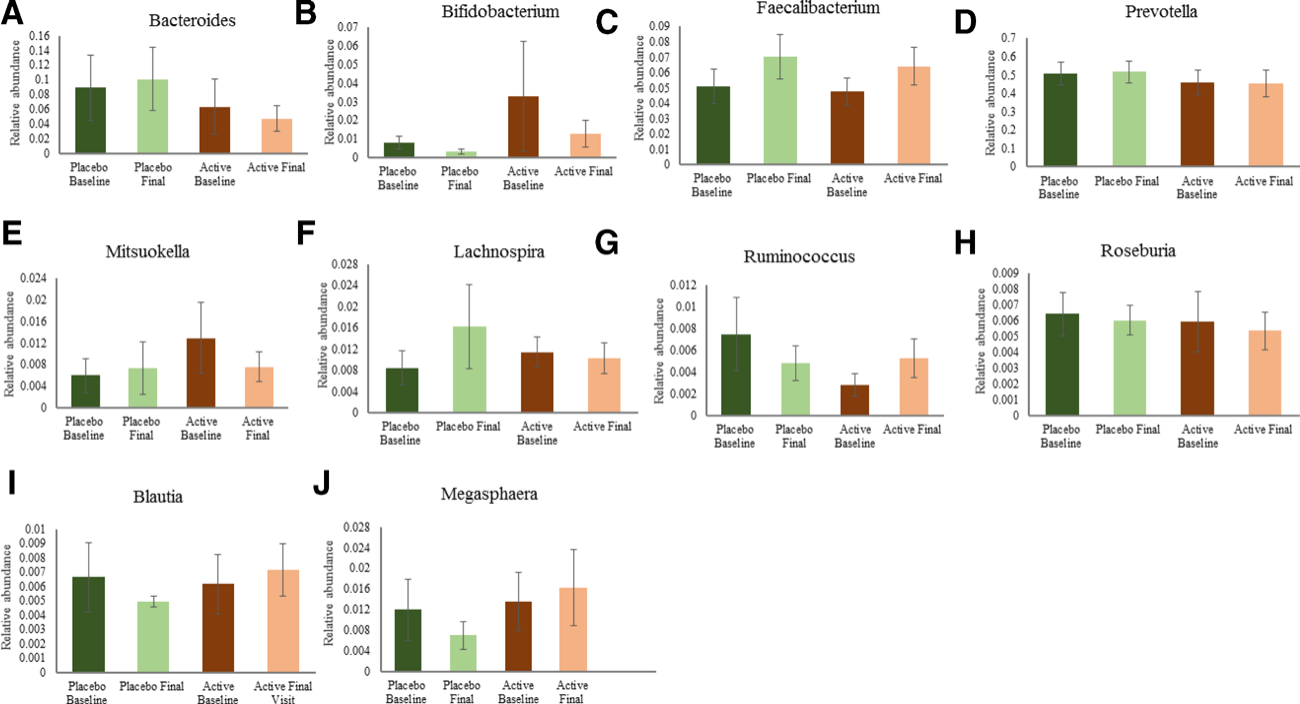
Relative abundance of bacterial genus (A) *Bacteroides*; (B) *Bifidobacterium*; (C) *Faecalibacterium*; (D) *Prevotella*; (E) *Mitsuokella*; (F) *Lachnospira*; (G) *Ruminococcus*; (H) *Roseburia*; (I) *Blautia*; (J) *Megasphaeara* in active and placebo groups at baseline and final visit. Values expressed as mean ± SEM.

A subtle change in the relative percentage of some genus was observed in both the groups which are represented in Figure 5. A percentage increase in relative abundance was observed in *Faecalibacterium* (34.42%), *Blautia* (16.04%), *Megasphaera* (19.74%), *Ruminococcus* (86.31%), and a decrease in abundance was found in *Bacteroides* (25.45%), *Roseburia* (9.69%), *Lachnospira* (9.56%), *Mitsuokella* (40.95%), *Bifidobacterium* (61.25%) and *Prevotella* (0.84%) in subjects supplemented with *B. coagulans* MTCC 5856.

In placebo, a percentage increase was observed in the genus *Bacteroides* (12.69%), *Prevotella* (1.56%), *Faecalibacterium* (37.36%), *Lachnospira* (91.11%) and *Mitsuokella* (22.17%), while *Roseburia* (6.01%), *Blautia* (25.63%), *Megasphaera* (41.57%), *Bifidobacterium* (57.91%), and *Ruminococcus* (35.75%) showed a percentage decrease in relative abundance when compared from baseline to final visit (Fig. 5A–J).

Pathogens like *Staphylococcus* species *C. coccoides, C. histolyticum*, and *Pseudomonas* were not observed in any of the samples. *Akkermansia muciniphila* and *Lactobacilli* species were observed but were significant only in a few samples with fewer read assignments. The microbiome group such as *Bacteroidetes, Bifidobacterium* spp., *Enterobacteriaceae*, and *Faecalibacterium prausnitzii* were observed across both active and placebo groups with *P* value significance. The species, *Bacteroides ovatus* was observed only in a few active baseline visit samples and not observed in other samples. Multiple *Lactobacilli* species, especially *Lactobacillus ruminis* subgroup, were observed in maximum samples across groups (Supplementary Table S1, Supplemental Digital Content, http://links.lww.com/MD/I987).

### 3.3. Enumeration of *B. coagulans* by RT-qPCR

*B. coagulans* was detected in all the fecal samples of active and placebo subjects at baseline and at the end of the study by quantitative real-time PCR technique. The details of primer design, standard curve for primer efficiency, and amplification plots are shown in Supplementary Fig. S3A–E, Supplemental Digital Content, http://links.lww.com/MD/I988.

**Figure 6.**
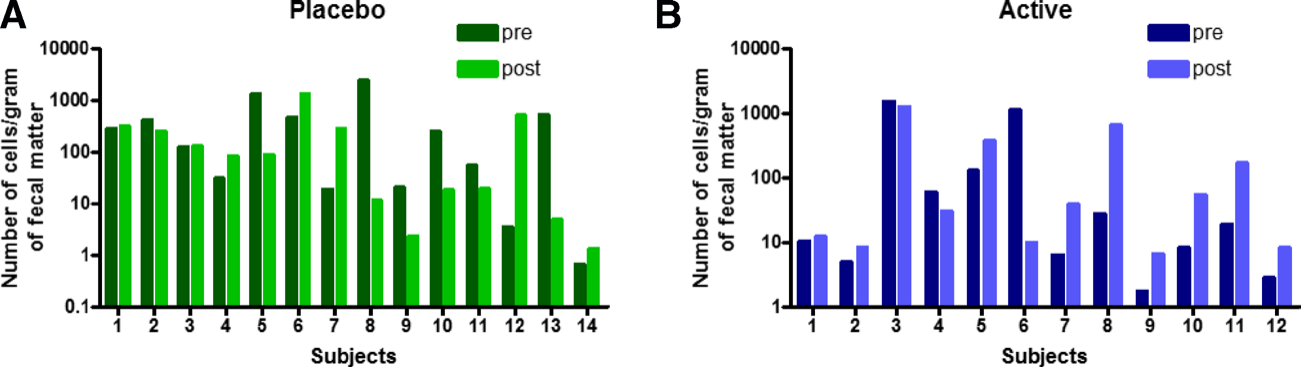
Enumeration of *Bacillus coagulans* by quantitative real-time PCR showed that fecal samples from the active group showed an increase in *B. coagulans* cell count compared to that of the placebo group. PCR = polymerase chain reaction.

We observed a wide variation in the cell count (14.88–191.69 cells per gram of fecal sample) in the baseline samples. Although we could recover and enumerate *B. coagulans* with a sensitivity of 10^−1^ to 10^4^ cells per gram of the sample using qPCR, the numbers were highly different in the 2 groups. In the active group, 9 out of 12 samples showed a relative increase in *B. coagulans* cell count, while in placebo, 5 out of 14 samples showed an increase in count (Fig. 6).

### 3.4. Clinical data on safety

**Figure 7.**
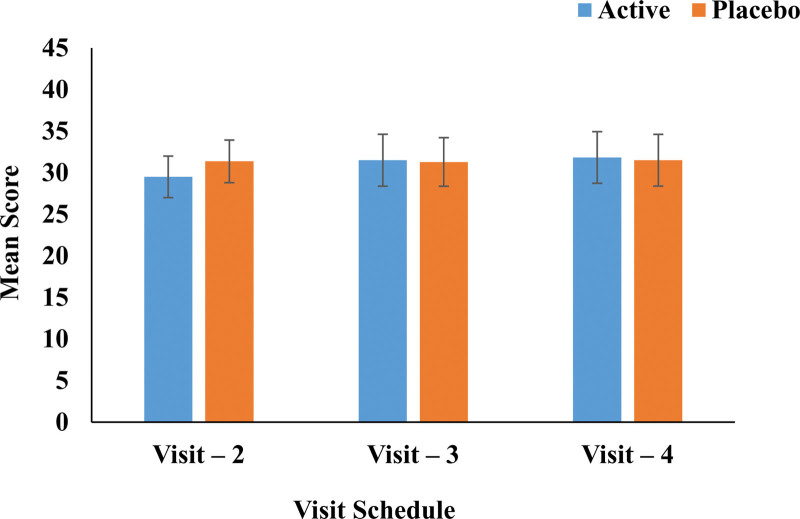
Abdominal Health Questionnaire Assessment analysis.

The vital signs, namely, blood pressure, respiratory rate, pulse rate, and biochemical parameters, were in the normal range in both active and placebo groups attesting to the safety of the *B. coagulans* MTCC 5856 consumption. Hematological, biochemical, and biomarkers like hs-CRP, IL-6, IL-10, and TNF-α did not show any significant change in active and placebo groups from baseline to the final visit (Table 2). None of the enrolled subjects had an abnormal medical history. There were no statistically and clinically significant changes in the body weight and body mass index from baseline to the last visit or between the treatment groups. There was no significant change in Abdominal Health Questionnaire from the baseline to the final visit. Intergroup comparison of the Active group against the placebo group showed no significant difference between the groups (Fig. 7). The Bristol Stool Chart for the active group was predominantly of type 5 at the last visit as compared to baseline and was of type 3 and 4 for the placebo group suggesting the stools were in the normal range for active and placebo at the baseline visit and final visit (Table 3).

**Table 2:** Summary of hematology, biochemistry, and biomarkers (mean ± SD) analysis.

Parameters	Active		Placebo		*P* value (placebo vs active at the end of the study)
	Baseline	Final visit	Baseline	Final visit	
Hemoglobin (g/dL)	14.23 ± 2.38	13.93 ± 2.79	13.98 ± 2.90	14.36 ± 2.55	.6851
Erythrocyte count (×10^6^/µL)	5.14 ± 0.54	5.08 ± 0.49	5.08 ± 0.63	5.09 ± 0.69	.9669
Platelet count (×10^3^/µL)	304.08 ± 70.99	302.25 ± 79.95	302.29 ± 88.13	315.07 ± 92.87	.7119
Leukocyte count (×10^3^/µL)	7.30 ± 2.45	7.65 ± 1.45	7.44 ± 1.64	7.96 ± 1.42	.5877
Packed cell volume (%)	44.75 ± 4.74	44.16 ± 5.34	43.40 ± 5.28	44.30 ± 4.83	.9446
Mean cell volume (fL)	88.76 ± 8.75	89.06 ± 11.08	89.69 ± 8.58	91.56 ± 7.01	.4919
Mean platelet volume (fL)	10.17 ± 0.62	10.33 ± 0.99	10.36 ± 0.59	10.47 ± 0.63	.6663
Mean corpuscular hemoglobin (pq)	27.68 ± 3.89	27.43 ± 4.79	27.61 ± 3.54	28.17 ± 2.60	.6219
Mean corpuscular hemoglobin concentration (g/dL)	31.08 ± 2.08	30.63 ± 2.53	30.85 ± 1.41	30.76 ± 1.36	.8691
Lymphocytes (%)	36.69 ± 8.14	32.33 ± 3.45	29.96 ± 7.47	31.94 ± 8.45	.8826
Eosinophils (%)	4.51 ± 3.47	5.02 ± 3.25	3.59 ± 2.32	3.73 ± 2.69	.2789
Monocytes (%)	2.78 ± 0.88	2.91 ± 0.66	2.99 ± 1.16	2.64 ± 0.96	.4197
Neutrophils (%)	55.60 ± 9.44	59.28 ± 5.82	62.99 ± 7.49	62.32 ± 7.94	.2838
Basophils (%)	0.18 ± 0.04	0.19 ± 0.03	0.19 ± 0.03	0.19 ± 0.05	1.0000
Total cholesterol (mg/dL)	191.83 ± 38.02	206.00 ± 39.13	181.93 ± 30.21	204.21 ± 29.80	.8958
LDL cholesterol (mg/dL)	124.50 ± 30.83	130.58 ± 37.61	112.93 ± 20.54	119.36 ± 19.70	.3401
Triglycerides (mg/dL)	133.67 ± 68.11	159.83 ± 84.81	166.21 ± 88.90	180.64 ± 74.25	.5109
HDL cholesterol (mg/dL)	46.17 ± 9.60	51.08 ± 10.66	46.21 ± 12.50	48.29 ± 10.16	.5015
Alkaline phosphate (U/I)	89.58 ± 29.45	91.90 ± 33.43	91.44 ± 21.28	89.33 ± 21.18	.8141
Total bilirubin (mg/dL)	0.64 ± 0.30	0.54 ± 0.18	0.56 ± 0.22	0.50 ± 0.24	.6399
SGOT (U/I)	31.94 ± 23.99	32.19 ± 24.52	27.23 ± 9.37	24.70 ± 9.39	.3002
SGPT (U/I)	22.65 ± 12.01	30.29 ± 18.52	24.34 ± 13.21	28.14 ± 20.26	.7815
Serum creatinine (mg/dL)	0.70 ± 0.16	0.72 ± 0.15	0.74 ± 0.15	0.77 ± 0.16	.4218
hs-CRP (mg/dL)	0.38 ± 0.72	0.50 ± 1.07	0.2 ± 0.49	0.2 ± 0.45	.3091
IL-10 (pg/mL)	1.00 ± 0.00	1.00 ± 0.00	1.00 ± 0.00	1.00 ± 0.00	N/A
TNF-α (pg/mL)	6.30 ± 5.64	8.94 ± 14.18	14.78 ± 35.27	8.82 ± 10.56	.9805

hs-CRP = high-sensitivity C-reactive protein, IL-10 = interleukin 10, TNF = tumor necrosis factor.

**Table 3:** Bristol stool chart of placebo and active group at baseline and final visit.

Types of stool	Placebo group (N = 14)		Active group (N = 12)	
	Baseline visit N (%)	Final visit N (%)	Baseline visit N (%)	Final visit N (%)
Type-1	0 (0.00)	0 (0.00)	0 (0.00)	0 (0.00)
Type-2	4 (28.57)	2 (14.29)	2 (16.67)	2 (16.67)
Type-3	3 (21.43)	5 (35.71)	3 (25.00)	1 (8.33)
Type-4	5 (35.71)	5 (35.71)	1 (8.33)	0 (0.00)
Type-5	1 (7.14)	0 (0.00)	4 (33.33)	7 (58.33)
Type-6	1 (7.14)	2 (14.29)	2 (16.67)	2 (16.67)
Type-7	0 (0)	0 (0)	0 (0)	0 (0)

N = Number of Subjects, Type 1 is indicative of separate hard lumps (hard to pass), Type 2: lumpy, sausage-shaped, Type 3: sausage-shaped with cracks on the surface, Type 4: sausage-shaped or snake-like; smooth and soft, Type 5: soft blobs with clear-cut edges (easy to pass), Type 6: fluffy pieces with ragged edges; mushy, Type 7: entirely liquid, watery, no solid pieces.

## 4. Discussion

The present study was carried out to understand the influence of probiotic supplementation on changes in the gut microbiota composition in healthy adults and to enumerate *B. coagulans* in the fecal samples. The metagenome analysis revealed no major changes in the gut microbiome composition in individuals supplemented with *B. coagulans* MTCC 5856. However, minor positive changes were observed in supplemented individuals. Further, using quantitative RT-PCR (qRT-PCR), we could detect and enumerate *B. coagulans* species in the fecal samples of the human subjects using qRT-PCR. However, the numbers showed considerable variations in each individual. The probiotic did not adversely affect the abdominal health, stool pattern, or clinical and biochemical parameters, reiterating its safety for human consumption.

The gut microbiome alters the gene expression of mammalian gut mucosa involved in immunity, metabolism, and nutrient absorption, which ultimately affects the functions of the GI tract. The diversity of the bacterial population in the gut is of critical importance in human health, as decreased microbiome diversity is correlated with several disease conditions.^[29]^ The gut microbiome diversifies with age, ethnicity, lifestyle, and dietary habits, and the loss of microbial diversity is associated with Crohn disease, IBS, and metabolic diseases.^[30–32]^ We observed a minor shift towards a higher microbial diversity in subjects supplemented with *B. coagulans* MTCC 5856.

Probiotics should have minimal influence on microbiota composition in the absence of dysbiosis.^[9,10,33]^ On the contrary, under conditions of dysbiosis, as in IBS, probiotic supplementation is reported to restore the gut microbiome to normalcy.^[21]^

The overall composition of the gut microbiome of both active and placebo group subjects was dominated by *Bacteroidetes, Firmicutes, Proteobacteria*, and *Actinobacteria*. The gut microbiota composition at the baseline and the final visit did not show a significant variation in the percentage abundance of *Bacteroidetes* and *Firmicutes*. However, a difference was observed in the *Proteobacteria* and *Actinobacteria*, in the probiotic-supplemented group. A load of proteobacteria is considered a potential diagnostic criterion for gut dysbiosis and disease.^[34]^ An abundance of *Proteobacteria* is associated with Crohn disease, while its decrease improves digestive health.^[35]^ In contrast, *Actinobacteria* are lower in IBS patients.^[31]^ We observed a decrease in the abundance of *Proteobacteria* and an increase in *Actinobacteria* in the *B. coagulans* MTCC 5856 supplemented subjects, suggesting a positive impact on gut microflora. Members of the *Cyanobacteria, Elusimicrobia, Lentisphaeraee, Tenericutes, Verrucomicrobia*, TM7, and *Fusobacteria* were also present in our study subjects in the gut, although their abundance was low. These results are in concurrence with reported studies on the abundance of these phyla in a healthy Indian population.^[36]^

At the genus level, a percentage increase was observed in *Faecalibacterium* in both groups. Ethnicity, lifestyle, food habits, exercise, stress, and various other factors influence gut microbiota composition. The predominance of genera belonging to *Prevotella* and *Megasphaera* was reported to be a distinctive feature of Indian gut flora.^[36]^ The anaerobic bacteria *F. prausnitzii* is one of the main commensal bacterium components of gut microbiota, belonging to the phylum *Firmicutes*, which is considered a bioindicator of human health. It is a butyrate-producing bacteria that protects the gut from inflammation.^[37]^ The depletion of its population is associated with intestinal disorders, especially Crohn disease.^[38]^ Further, patients suffering from intestinal and metabolic disorders such as IBD, colorectal cancer, obesity, and celiac disease were reported to have lower levels of *F. prausnitzii*.^[36,39–41]^ By increasing the abundance of *F. prausnitzii, B. coagulans* MTCC 5856 may benefit individuals predisposed to chronic metabolic and intestinal disorders. Consistent with our results, a recent study showed upregulation of *Actinobacteria* and *Firmicutes* and downregulation of *Bacteroids, Proteobacteria, Streptophyta*, and *Verrucomicrobia* in IBS patients treated with *B. coagulans* LBSC.^[21]^ *Blautia, Megasphaera*, and *Ruminococcus* were the other genera that showed an increase in the consumption of *B. coagulans* MTCC 5856. *Blautia* is a *Firmicute* of the *Lachnospiraceae* family which can degrade complex polysaccharides in the diet to produce short-chain fatty acids (SCFA), providing energy to the host.^[41]^ It is also widely present in the Indian population and is reported to be inversely associated with visceral fat accumulation^[41,42]^ Interestingly, in the recent study on titanium dioxide nanoparticle-induced intestinal damage in obese mice, *B. coagulans* MTCC was found to increase the relative abundance of *Blautia* and alleviate intestinal damage.^[43]^ The SCFA produced by gut bacteria has several positive influences on the gut, like energy utilization, gut motility, and intestinal secretion.^[44,45]^ These fatty acids, especially butyrate, suppress inflammation, improves insulin secretion, and reduce adiposity and inflammation.^[46,47]^ *B. coagulans*, by themselves, are also known to produce SCFA.^[48]^

Previous reports regarding the ability of probiotic microorganisms to survive the gastrointestinal environment have been contradictory. While a few studies have reported successful recovery of various probiotic organisms from the feces following oral consumption,^[49,50]^ others have demonstrated a poor recovery of live organisms.^[51,52]^ These inconclusive results could be because the recovery of probiotics depends on the number of bacteria consumed, their resistance to acidity and bile salt, product composition, and the techniques used to identify the bacteria. qRT-PCR enables sensitive detection of bacterial species.^[53,54]^ Although we were able to detect and enumerate *B. coagulans*, albeit not the specific strain, we observed a huge variation in the numbers in individual samples.

*Bacillus* species are not believed to be natural inhabitants of the gut. They are transient organisms that make their way into the gut through the consumption of fermented food and vegetables. *B. coagulans*, being a spore-former, can survive in the stomach and germinate in the intestine.^[48]^ Several earlier studies have shown that the species has a poor ability to colonize in the intestinal tract of mammals.^[55]^ However, a recent in vitro study demonstrated the adherence of the *B. coagulans* MTCC 5856 spores to colonic cells.,^[56]^ while *B. coagulans* were shown to proliferate in the rat intestine temporarily.^[57]^ Using the sensitive qRT-PCR method, we could detect and enumerate *B. coagulans* species in our study population. However, in the metagenome data, its relative abundance was too low to detect. It is also well known that Metagenomics can detect bacteria only at concentrations of >10^5^ bacteria per gram.^[58]^ The present study provides confirmation that *B. coagulans* MTCC 5856 does not induce any drastic shift in the diversity or abundance of microbiome in healthy adults, but its consumption is safe and well tolerated. The microbiome of a healthy gut is considered relatively stable and it may resist the growth of other species even after probiotic supplementation. These results demand significant space in the growing body of probiotic research as the majority of probiotic consumers are healthy adults. Even though the probiotic *B. coagulans* MTCC 5856 has no significant impact on healthy individuals, it may be a safe preventive therapy for maintaining general health.

The study has some limitations. The randomization of the study was purely based on the subject demographic characteristics. The baseline microbiome analysis was not carried out before randomization, which could explain the difference in microbiome data between placebo and probiotic-supplemented groups. The study was conducted with a relatively low number of participants for a short time, resulting in a higher variation in the number of spores in the 2 groups. The study did not include a washout period post-supplementation. Further studies with different doses in a population with gut dysbiosis will help us ascertain the positive effect of this strain on gut microbiota composition.

## 5. Conclusion

The present study confirms that LactoSpore (*B. coagulans* MTCC 5856) is a safe probiotic, maintains gut health, and general health parameters in the normal range, and does not grossly alter the gut microbiota composition in healthy humans, thereby maintaining the delicate host-microbe relationship in the gut ecosystem. Another important observation from this study is the modulation of a few genera of *Firmicutes (Blautia* and *F. prausnitzii*), by supplementation, which is associated with an increase in SCFA production, reducing gut inflammation, and maintaining general health. These results warrant further studies on the effect of *B. coagulans* MTCC 5856 supplementation on microbiome modulation in individuals with gut dysbiosis. Future studies on the long-term consumption of the probiotic in a population with gut dysbiosis would be helpful in determining the role of *B. coagulans* MTCC 5856 supplementation in modulating the gut microbiome and its correlation with health benefits.

## Acknowledgments

We thank the clinical trial investigators and their team Divakars Specialty Hospital, Bangalore, members for conducting this clinical trial. The investigators and the team had no influence on any aspect relevant to this study. We thank Genotypic Technology Private Ltd., Bangalore team for sample processing, bioinformatics data analysis, and helpful discussion.

## Author contributions

Conceptualization: Muhammed Majeed.

Data curation: Sudha Rao.

Formal analysis: Sudha Rao, Lakshmi Mundkur.

Investigation: Hema Divakar, Sudha Rao.

Methodology: Kalyanam Nagabhushanam, Sivakumar

Arumugam.

Project administration: Hema Divakar, Sivakumar Arumugam.

Resources: Muhammed Majeed.

Supervision: Shaji Paulose, Hema Divakar, Sudha Rao.

Validation: Lakshmi Mundkur, Shaji Paulose.

Writing – original draft: Lakshmi Mundkur.

Writing – review & editing: Muhammed Majeed, Kalyanam Nagabhushanam, Lakshmi Mundkur, Shaji Paulose, Hema Divakar, Sudha Rao, Sivakumar Arumugam.

## Supplementary Material
