# Supplementary material for: Probiotic modulation of gut microbiota by Bacillus coagulans MTCC 5856 in healthy subjects: A randomized, double-blind, placebo-control study
Source: Medicine (Baltimore). 2023 May 17;102(20):e33751. doi: 10.1097/MD.0000000000033751 (PMC10194586; doi:10.1097/MD.0000000000033751)
Supplement: Supplementary file 1 [file medi-102-e33751-s001.pdf]

Probiotic modulation of gut microbiota by *Bacillus coagulans* MTCC 5856 in healthy subjects: A randomized, double-blind, placebo-control study

Muhammed Majeed PhD[1,2], Kalyanam Nagabhushanam PhD[2], Lakshmi Mundkur PhD[1], Shaji Paulose M. Pharm[1], Hema Divakar MD [3], Sudha Rao PhD [4] & Sivakumar Arumugam PhD [1]*

[1] Sami-Sabinsa Group Limited, 19/1, 19/2, First Main, Second Phase, Peenya Industrial Area Bangalore 560058, Karnataka, India

[2] Sabinsa Corporation, 20 Lake Drive, East Windsor, New Jersey, 08520, USA.

[3]Divakars Speciality Hospital, Bangalore 560078, Karnataka, India

[4] Genotypic Technology Private Limited, Bangalore 560094, Karnataka, India

**Corresponding Author*

S. Arumugam, Sami-Sabinsa Group Limited, 19/1 and 19/2, First Main, Second Phase, Peenya Industrial Area, Bangalore 560058, Karnataka, India, E-mail: sivakumar@sami-sabinsagroup.com

Supplementary Methods

Clinical Study Design

The study was conducted as a randomized, double-blind, placebo-controlled clinical trial. Thirty (30) healthy subjects were enrolled who were willing to adhere to the protocol criteria and who meet all the inclusion criteria and none of the exclusion criteria.

Inclusion criteria: Male and female adult healthy volunteers, aged between 25-55 years and BMI between 20-27 kg/m^2 (both inclusive), mixed diet consuming non-smokers, willing to come for regular follow up visits and, avoid the prebiotic and probiotic food supplements, laxatives and foods having laxative effects. Subjects able to give written informed consent.

Exclusion criteria: No gastrointestinal complaints like colonic irritation, not taking any medication with gastrointestinal activity like laxatives, had not taken antibiotics for 3 months before starting the study, consumption of yogurt, curd, prebiotic or probiotic supplements as part of their daily diet, not participated in a clinical study during the preceding 90 days and not willing to abide by the study procedures or not willing to provide stool samples for the study. Pregnancy and lactating, presently suffering from any inflammatory disorders and mental illness, and history of drug or alcohol abuse in the last 6 months were also considered. Randomization of subjects was done to receive either *B. coagulans* MTCC 5856 or Placebo. Subjects were administered one capsule of *B. coagulans* MTCC 5856 or Placebo orally once daily after food at the night. (Fig.1)

Randomization

Patients (N=30) were randomized into two groups, active and placebo. The study groups, investigators, and other staff were blinded to the group assignment.

Laboratory assessments

Subjects visited four times during the study duration, i.e., screening (-3days), baseline -visit 2 (day 0), visit

3 (day 14), and final visit (day 28). Vital signs, including blood pressure, respiratory rate, pulse rate, physical examination, were measured at all 4 visits and any abnormal lab/diagnostic parameters were considered for safety evaluations. Subject's demographics were recorded at screening and final visits. Medical and medication history was documented at the screening visit. The routine laboratory parameters of safety, i.e., hematology, lipid profile, serum biochemistry, human immunodeficiency virus, hepatitis B-virus, and hepatitis C-virus, 12- lead electrocardiogram were measured using standard laboratory techniques at screening and final visit. Urine test for pregnancy was performed on female volunteers of childbearing potential at screening and final visit. Adverse effects, if any, were recorded at each study visit (Table 1). All the tests were carried out as per the standard protocol.

For the analysis of serum biomarkers, hsCRP, IL-10, and TNF-α blood samples were collected from subjects at baseline and final visit. The serum biomarker test were carried out using ELISA following protocols from the manufacture.

Subject Abdominal Health Questionnaire and the Bristol Stool Chart were assessed at baseline and final visit.

For RT-PCR and 16S Illumina based sequencing Next Generation Sequencing (NGS), fecal samples were collected before dosing baseline and final visit (Table 1). All the fecal samples collected from subjects in fecal Collection Tube DNA/RNA Shield™ (Zymo Research) were stored at -80°C for prolonged storage.

Enumeration of *Bacillus coagulans*

RNA extraction for the rRNA-targeted reverse transcription-quantitative PCR (RT-qPCR)

The RNA extraction from the fecal samples is optimized using a standard Trizol method, Qiagen RNAeasy kit column, and NucleoSpin RNA stool kit. The RNA extracted using the NucleoSpin RNA stool kit was found to be optimal for the qPCR process.

RNA was isolated using a Nucleospin Fecal RNA kit, following the guidelines as per manufacturer guidelines. Briefly, the thawed sample was resuspended in a solution containing 346.5 of RLT buffer (Qiagen Sciences, Germantown, MD), 3.5µL of mercaptoethanol (Sigma-Aldrich Co.St. Louis, MO), and 100µL of Tris-EDTA buffer. Glass beads (300 mg; diameter,0.1 mm) (BioSpec Products, Inc., Bartlesville, OK) were added to the suspension, and the mixture was vortexed vigorously for 60 s using a Fast Prep FP 120 (BIO101, Vista, CA) at a power level of 5.0. Acid phenol (500µL, Wako Pure Chemical Industries, Ltd.) was added, and the mixture was incubated for 10 min at 60°C. After incubation, the mixture was cooled on ice for 5 min and added to 100µL of chloroform: isoamyl alcohol (24:1). After centrifugation at 12,000xg for 10 min at 4°C, 450µL of supernatant was collected and added to an equal volume of chloroform, isoamyl-alcohol. After centrifugation at 12,000xg for 5 min, 400µL of supernatant was collected and subjected to isopropanol precipitation. Finally, the nucleic acid fraction was suspended in 1 mL of nuclease-free water (Ambion, Inc., Austin, TX). The following DNase treatment was skipped in this study, because we confirmed that untreated and DNase-treated samples showed identical results in the preliminary experiments, indicating that contaminating DNA does not affect RT-qPCR quantification. The extracted RNA was quantified, and quality was assessed by Tape station and Bioanalyser.

16S rRNA specific primer design

Primer was designed by using 16S rRNA sequences obtained from the NCBI database for the Bacillaceae family and constructed multiple alignments of the target groups and reference *Bacillus coagulans* 16S

rRNA with the ClustalW program. The ClustalW alignment is shown in Supplementary Fig.1. After comparing the sequences, potential primer target sites were identified for specific detection of *Bacillus coagulans*. The designed primer specificity was assessed by performing primer BLAST against the Non-Redundant database by submitting the sequences to the NCBI Primer-BLAST program.

Establishment of an analytical system for the human fecal microbiota by Quantitative Reverse Transcription PCR

RT-qPCR was conducted in a one-step reaction using Super Script IV One-Step RT-PCR method. The methodology involves the use of 16S *B. coagulans* specific reverse primer for the cDNA conversion and its direct detection by standard SYBR Green chemistry. Briefly, 10ng of the extracted RNA was taken in a tube containing 1 µL of 2 mM 16S rRNA reverse primer, and the RNA-primer mix was incubated at 65°C for 5 minutes and chilled on ice for 1 minute. The reverse transcriptase mix containing 4 µL of 5X SSIV buffer, 1 µL of 100mM DTT, 1 µL of Ribonuclease inhibitor and 1 µL of SuperScript IV reverse transcriptase (200U/µL) was added. The reaction mix was incubated at 55°C for 15 minutes. Further, reverse transcriptase is inactivated by incubating the tubes at 80°C for 10 min. For qPCR reaction, 2 µL of the cDNA is used, and the Agilent Brilliant SYBR Green dye is used for the amplification detection. All qPCR reactions were performed using the Agilent Stratagene 3005 system. Total RNA fractions extracted from the vegetative and sporulating bacterial cells at a dose corresponding to 10^5 cells were assessed by RT-qPCR. Using the standard curve for the seven serial dilutions was analyzed to check the efficiency of primer, and the same is used against all test samples to estimate the number of *Bacillus coagulans* cells. The amplified signal was judged as positive when it was more than that of 10^1 standard cells and as negative when less than that of 10^{-1} standard cells. The cell count

between 10^{-1} -10^{4} against the standard curve was defined as positive for *Bacillus coagulans*. The final cell count was estimated by the formula-

Total Cells = Number of Cells determined by Standard curve* (Total yield/Total Weight of fecal matter used for extraction)

Metagenome Sequencing

DNA extraction for the metagenome sequencing

A fecal sample (approximately 500µL) was taken in a sterile 1.5mL vial, and 300µL (10mg/mL) of lysozyme (Sigma # L6876) was added. The sample tube was inverted mixed and incubated at room temperature for 30 minutes at 37°C. To this, 200µL of AL buffer was added and vortexed. Samples were subjected to Proteinase K treatment at 56°C for 2 h, followed by RNase treatment at 65°C for 20 min. The lysate was mixed well with 100% alcohol and loaded onto Qiagen DNeasy blood and tissue column (#69506). DNA was purified by the following steps provided in the manufacturer's guidelines. Finally, DNA was eluted in 1X TE buffer and stored at -20°C freezer. DNA was quantified by Nanodrop2000 and analyzed on 0.8% agarose gel.

Library preparation and sequencing

Sequencing libraries were constructed by a two-step PCR-based workflow described as follows: 1. Round 1 PCR: The 16S rRNA gene V3-V4 regions were first amplified using region-specific proprietary primers developed at Genotypic Technology Pvt. Ltd., Bangalore, India, Briefly, using KAPA HiFi Hot Start PCR Kit (KAPA Biosystems Inc., Boston, MA USA), and 5µM primer concentration, 50ng of genomic DNA was amplified for 26 cycles. The amplicons thus generated were analyzed on 1.2% agarose gel.

2. Round 2 PCR for indexing: 1µL of 1:2 diluted round 1 PCR amplicons were amplified for 10 cycles to add Illumina sequencing barcoded adaptors (Nextera XT v2 Index Kit, Illumina, U.S.A.). Round 2 PCR amplicons (sequencing libraries) were analyzed on 1.2% agarose gel. The libraries were normalized and pooled for multiplex sequencing. Finally, these pools were quantified using Qubit dsDNA HS assay and fluorometer (Thermo Fisher Scientific, MA, USA) and then diluted to 2nM final concentration using Resuspension Buffer (RSB-Illumina, CA, USA). The normalized sample was denatured for 5 minutes using 0.2 N NaOH and neutralized by HT1 Buffer (Illumina, CA, USA). Denatured libraries were further diluted 13pM concentration for loading. Samples were then loaded into an Illumina MiSeq v3 600 cycles cartridge (Illumina, CA, USA). The flow cell and the PR2 buffer were placed in the designated slots in the machine and the run was performed in paired-end mode with 275 bp read length for each of forward (Read 1) and reverse (Read 2) reads. After the completion of the sequencing run, the data were demultiplexed using bcl2fastq software v 2.20 and Fast Q files were generated based on the unique dual barcode sequences. The sequencing quality was assessed using Fast QC v0.11.8 software. The adapter sequences were trimmed and bases above Q30 were considered and low-quality bases were filtered off during reading pre-processing and used for downstream analysis.

Metagenome analysis

From the Illumina paired-end raw reads of 52 samples, the reads having a V3-V4 primer sequence and high-quality bases were filtered. Short overlapping forward and reverse reads coming from the same fragment were joined using Fastq-join[24] to form sequences of the V3-V4 hypervariable 16S rRNA region. These stitched reads were considered for microbiome search using the QIIME[25] pipeline. The query sequences were clustered using the UCLUST[26] method against a curated chimera free 16s rRNA database (Greengenes[27]v 13.8). The taxonomies were assigned using the RDP[28] classifier to these

clusters at >=97% sequence similarity against the reference database, which resulted in the generation of a biom file that was further used for advanced analysis and visualization. The biom file contains information about the number of reads assigned to particular taxa. The details such as reads utilized in the identification of microbiome and the number of OTUs picked for each sample were identified using QIIME scripts. Relative abundance from phylum to species was calculated from read counts assigned to OTU is divided by total utilized reads for microbiome search. The biom was utilized further for advanced analysis and visualization. The filtered rarefied biom at depth of 22,000 sequences/sample was used for the calculation of alpha diversity indices using various metrices i.e., Shannon, Simpson, chao1 and beta-diversity.

Comparative analysis across a group of samples

The comparative analysis was performed by two ways to identify the abundance of specific species in the samples. From the 52 samples, the samples belong to the active and placebo groups are compared. This comparison was performed by considering the difference of relative abundance between baseline and final visit samples to compare between active and placebo groups. Further, the baseline and final visit samples of active group and placebo group were compared. This comparison provides a difference in taxonomy abundance between baseline and final visit of samples. The main focus of the comparative analysis was to quantify some of the bacterial species that predominate the human intestine (*Clostridium coccoides* group, *Clostridium Leptum* subgroup, *Bacteroides fragilis* group, *Bifidobacterium* group, *Atopobium* cluster, *Eubacterium recale-C-histolyticum* subgroup and *Prevotella*), eight potential pathogens (*Clostridum difficile, Clostridium perfringens, Enterobacteriaceae, Enterococcus* spp., *Streptococcus* spp., *Staphylococcus* spp., *Escherichia coli and Pseudomonas* spp.,) and few Lactobacilli

groups (*L. gasseri* subgroup, *L. brevis, L. casei* subgroup, *L. fermentum, L. fructivorans, L. plantarum* subgroup, *L. reuteri* subgroup, *L. ruminis* subgroup and *L. sakei* subgroup).

Bioinformatics analysis

Alpha diversity was calculated using different matrices i.e. Shannon, Simpson, Chao1 and observed species. Beta diversity was determined by principal coordinate analysis using unweighted and weighted UniFrac metrics. Emperor 3D viewer was used to visualize the plots. To determine the statistical significance between the two groups, taxon differential abundance across the groups was performed in QIIME (QIIME: group significance.py) to examine whether observation counts (i.e., OTUs and Microbial taxon) are significantly different between the groups (eg., Baseline visit, and Final visit). The OTU table prior to final community quality control was collapsed at each taxonomic level (i.e., Phylum–nus; QIIME: collapse_taxonomy.py), with counts representing the relative abundance of each microbial taxon differences in the mean abundance of taxa between sample groups were calculated using Kruskal-Wallis nonparametric statistical tests. The taxa were ranked with p-values of most to least significant (p<.05) are provided alongside false discovery rate and Bonferroni corrected p-values, and then the taxon was ranked from most to least significant (p<.05).
