# Supplementary material for: Probiotic modulation of gut microbiota by Bacillus coagulans MTCC 5856 in healthy subjects: A randomized, double-blind, placebo-control study
Source: Medicine (Baltimore). 2023 May 17;102(20):e33751. doi: 10.1097/MD.0000000000033751 (PMC10194586; doi:10.1097/MD.0000000000033751)
Supplement: Supplementary file 4 [file medi-102-e33751-s004.pdf]

Table S1. Relative Abundance significantly different taxa between active and placebo group.

Taxanomy						OTU	Active Vs Placebo P value (Kruskalwallis)	Active Samples			Placebo samples		
Phyllum	Class	Order	family	genus	species			Average	SD	No of samples	Average	SD	No of samples
		***Clostridium* spp.**											
Firmicutes	Clostridia	Clostridiales	Veillonellaceae			4300127	0.00273	0.0730	0.2034	18	0.0071	0.0248	6
Firmicutes	Clostridia	Clostridiales	Clostridiaceae			173921	0.00523	0.0040	0.0084	7	0.0001	0.0004	1
Firmicutes	Clostridia	Clostridiales	Lachnospiraceae	*Blautia*		177905	0.00591	0.0016	0.0014	8	0.0002	0.0004	2
Firmicutes	Clostridia	Clostridiales	Veillonellaceae	*Mitsuokella*	*multacida*	13811	0.01388	0.6604	1.4367	20	0.0149	0.0192	18
Firmicutes	Clostridia	Clostridiales	Lachnospiracea	*Lachnospira*		314095	0.01427	0.0542	0.0698	20	0.0184	0.0393	11
Firmicutes	Clostridia	Clostridiales	Ruminococcace	*Rumino coccus*		193336	0.01769	0.0056	0.0072	12	0.0050	0.0135	9
Firmicutes	Clostridia	Clostridiales	Clostridiaceae	*Sarcina*		300662	0.02163	0.0008	0.0013	4	0.0000	0.0000	0
Firmicutes	Clostridia	Clostridiales	Clostridiaceae	*SMB53*		345322	0.02390	0.0000	0.0000	0	0.0004	0.0006	5
Firmicutes	Clostridia	Clostridiales	Mogibacteriacea			3805726	0.02390	0.0000	0.0000	0	0.0008	0.0012	5
Firmicutes	Clostridia	Clostridiales				583958	0.02461	0.0024	0.0041	13	0.0040	0.0046	17
Firmicutes	Clostridia	Clostridiales	Veillonellaceae			820764	0.02552	0.1433	0.2431	11	0.0005	0.0015	2
Firmicutes	Clostridia	Clostridiales	Lachnospiraceae	*Coprococcus*		842596	0.02614	0.0054	0.0130	11	0.0007	0.0011	6
Firmicutes	Clostridia	Clostridiales	Ruminococcaceae			191582	0.03161	0.0177	0.0205	11	0.0065	0.0122	11
Firmicutes	Clostridia	Clostridiales	Ruminococcaceae	*Faecalibacterium*	*prausnitzii*	365842	0.04616	0.0021	0.0030	10	0.0010	0.0014	8
Firmicutes	Clostridia	Clostridiales	Lachnospiraceae			577206	0.04800	0.0015	0.0016	9	0.0006	0.0009	7
Firmicutes	Clostridia	Clostridiales	Lachnospiraceae	*Roseburia*		3152469	0.04840	0.0000	0.0000	0	0.0006	0.0012	4
Firmicutes	Clostridia	Clostridiales	Clostridiaceae	02d06		181342	0.04840	0.0000	0.0000	0	0.0003	0.0005	4
		Enterobacteriaceae											
Proteobacteria	Gamma proteo bacteria	Entero bacteriales	Enterobacteriaceae			166908	0.051120	0.0003	0.0005	3	0.0000	0.0000	0
Proteobacteria;	Gamma proteo bacteria	Entero bacteriales	Entero bacteriaceae	*Trabulsiella*		687792	0.051120	0.0004	0.0008	3	0.0000	0.0000	0
Proteobacteria;	Gamma proteo bacteria	Entero bacteriales	Entero bacteriaceae	*Erwinia*	*soli*	4455895	0.051508	0.0005	0.0009	4	0.0000	0.0000	0
		Enterococcus											
Firmicutes	Bacilli	Lactobacillales	Entero coccaceae	*Enterococcus*		1111582	0.28009	0.0002	0.0007	1	0.0000	0.0000	0
		Escherichia coli											
Proteobacteria	Gamma proteo bacteria	Entero bacteriales	Entero bacteriaceae	*Escherichia*	*coli*	114510	0.10343	0.0033	0.0029	14	0.0024	0.0050	7
Firmicutes	Erysipelo trichi	Erysipelo trichales	Erysipelo trichaceae	*Eubacterium*	*dolichum*	548587	0.09456	0.0000	0.0000	0	0.0004	0.0009	4
Firmicutes	Erysipelo trichi	Erysipelo trichales	Erysipelo trichaceae	*Eubacterium*		815422	0.35454	0.0000	0.0000	0	0.0001	0.0003	1
Firmicutes	Erysipelo trichi	Erysipelo trichales	Erysipelo trichaceae	*Eubacterium*	*biforme*	524884	0.49710	0.0959	0.2405	16	0.0130	0.0173	21
		Faecalibacterium prausnitzii											
Firmicutes	Clostridia	Clostridiales	Ruminococcaceae	*Faecalibacterium*	*prausnitzii*	189092	0.01224	0.0003	0.0007	4	0.0028	0.0056	12
		***Lactobacillus* spp.**											
Firmicutes	Bacilli	Lactobacillales	Lactobacillaceae	*Lactobacillus*	*reuteri*	411486	0.28009	0.0001	0.0003	1	0.0000	0.0000	0
Firmicutes	Bacilli	Lactobacillales	Lactobacillaceae	*Lactobacillus*	*mucosae*	4358602	0.45746	0.0002	0.0004	2	0.0001	0.0003	1
Firmicutes	Bacilli	Lactobacillales	Lactobacillaceae	*Lactobacillus*		807795	0.51629	0.0005	0.0013	2	0.0039	0.0144	2
Firmicutes	Bacilli	Lactobacillales	Lactobacillaceae	*Lactobacillus*	*ruminis*	178213	0.53682	0.4276	0.8068	21	0.1918	0.3608	27
Firmicutes	Bacilli	Lactobacillales	Lactobacillaceae	*Lactobacillus*	*iners*	130864	0.86745	0.0086	0.0298	1	0.0001	0.0003	1
		Prevotella											
Bacteroidetes	Bacteroidia	Bacteroidales	Prevotellaceae	*Prevotella*	*stercorea*	2220494	0.00650	0.0489	0.0621	16	0.0075	0.0163	6
Bacteroidetes	Bacteroidia	Bacteroidales	Prevotellaceae	*Prevotella*	*copri*	328035	0.04938	0.3021	0.6457	18	0.0101	0.0158	16
		Akkermansia											
Verruco microbia	Verruco microbiae	Verruco microbiale	Verruco microbiaceae	*Akkermansia*	*muciniphila*	363731	0.48891	0.0113	0.0259	5	0.1306	0.4047	7
