# Supplementary material for: Probiotic modulation of gut microbiota by Bacillus coagulans MTCC 5856 in healthy subjects: A randomized, double-blind, placebo-control study
Source: Medicine (Baltimore). 2023 May 17;102(20):e33751. doi: 10.1097/MD.0000000000033751 (PMC10194586; doi:10.1097/MD.0000000000033751)
Supplement: Supplementary file 5 [file medi-102-e33751-s005.pdf]

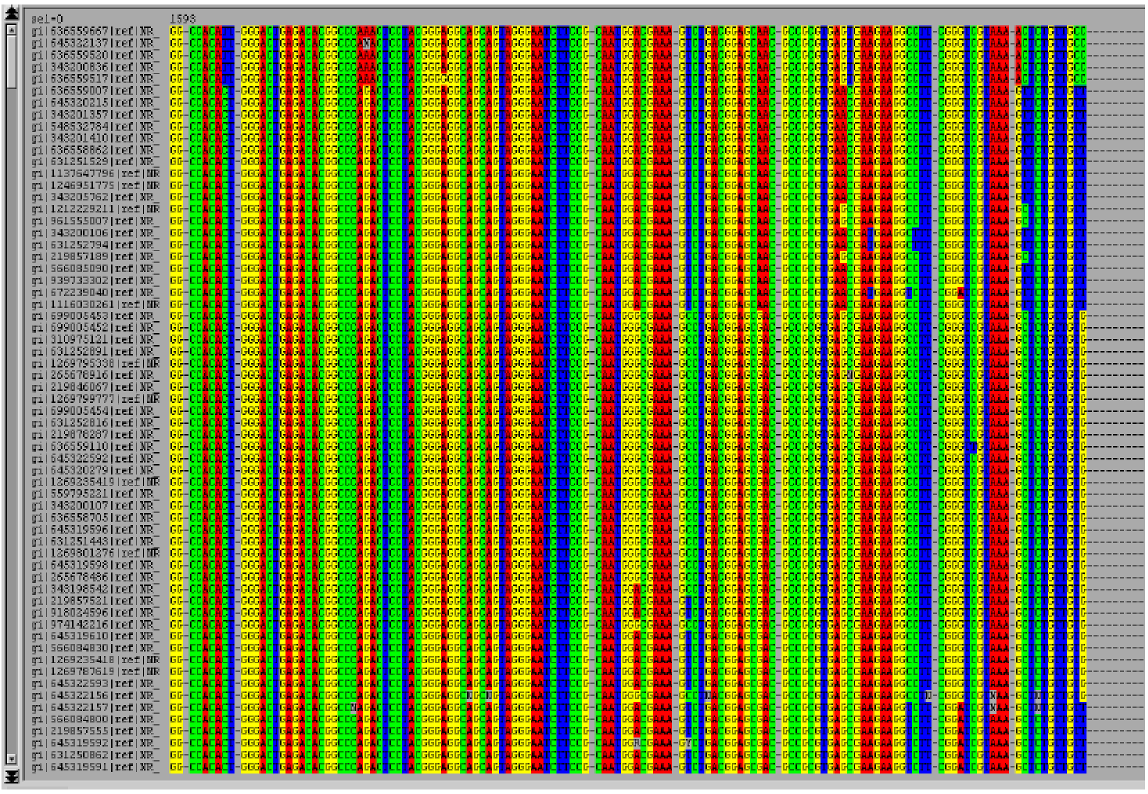

Figure S3A: Clustal W alignment view for the 16S rRNA region of Bacillaceae family.

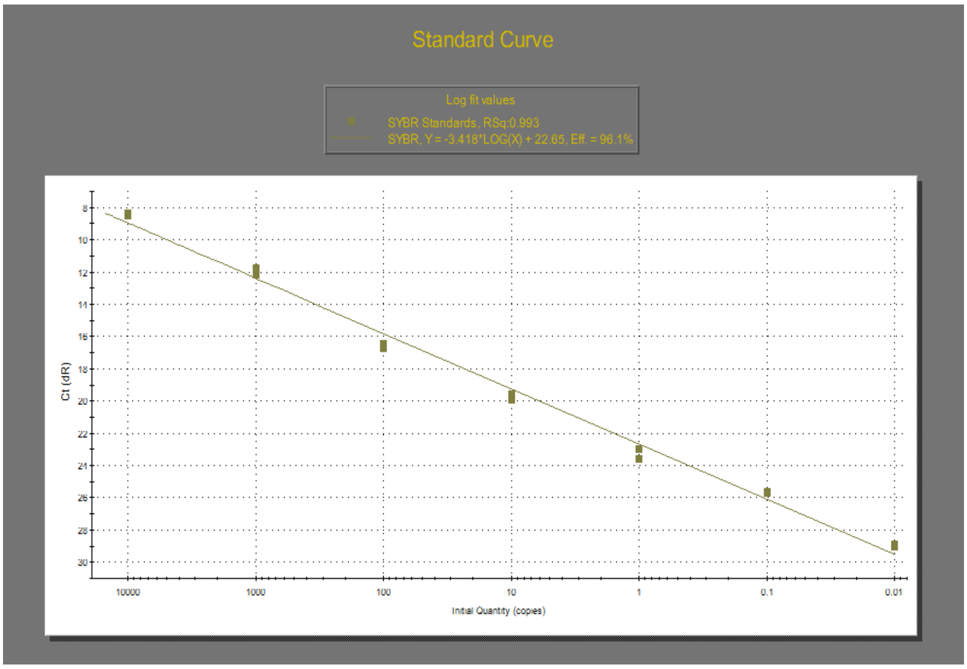

Figure S3B: Standard curve *B. coagulans* primer efficiency and validation.

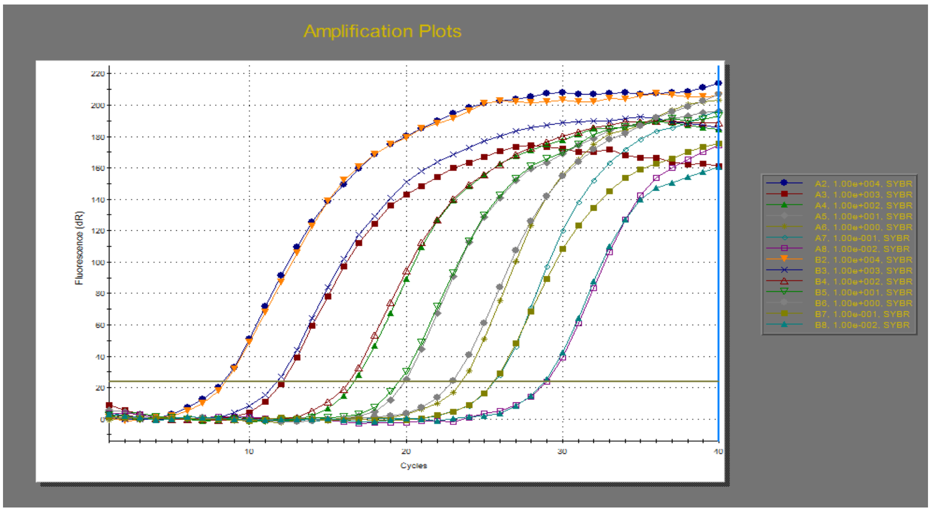

Figure S3C: *B. coagulans* cells: PCR – amplification plots.

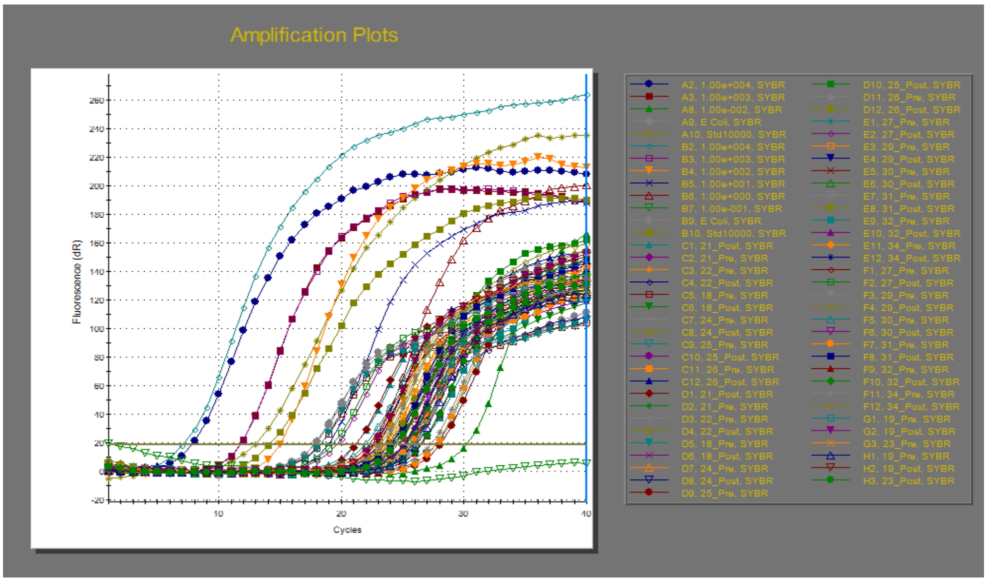

Figure S3D: PCR – Amplification plots of sample and standard.

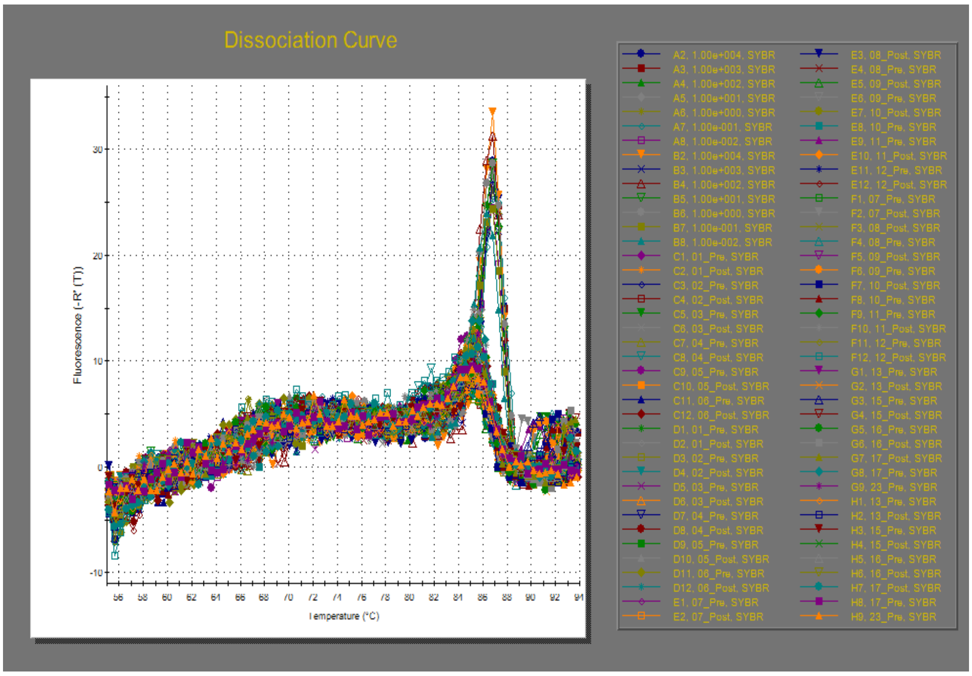

Figure S3E: PCR – Dissociation curve of sample and standard.
