# Supplementary material for: Probiotic modulation of gut microbiota by Bacillus coagulans MTCC 5856 in healthy subjects: A randomized, double-blind, placebo-control study
Source: Medicine (Baltimore). 2023 May 17;102(20):e33751. doi: 10.1097/MD.0000000000033751 (PMC10194586; doi:10.1097/MD.0000000000033751)
Supplement: Supplementary file 3 [file medi-102-e33751-s003.pdf]

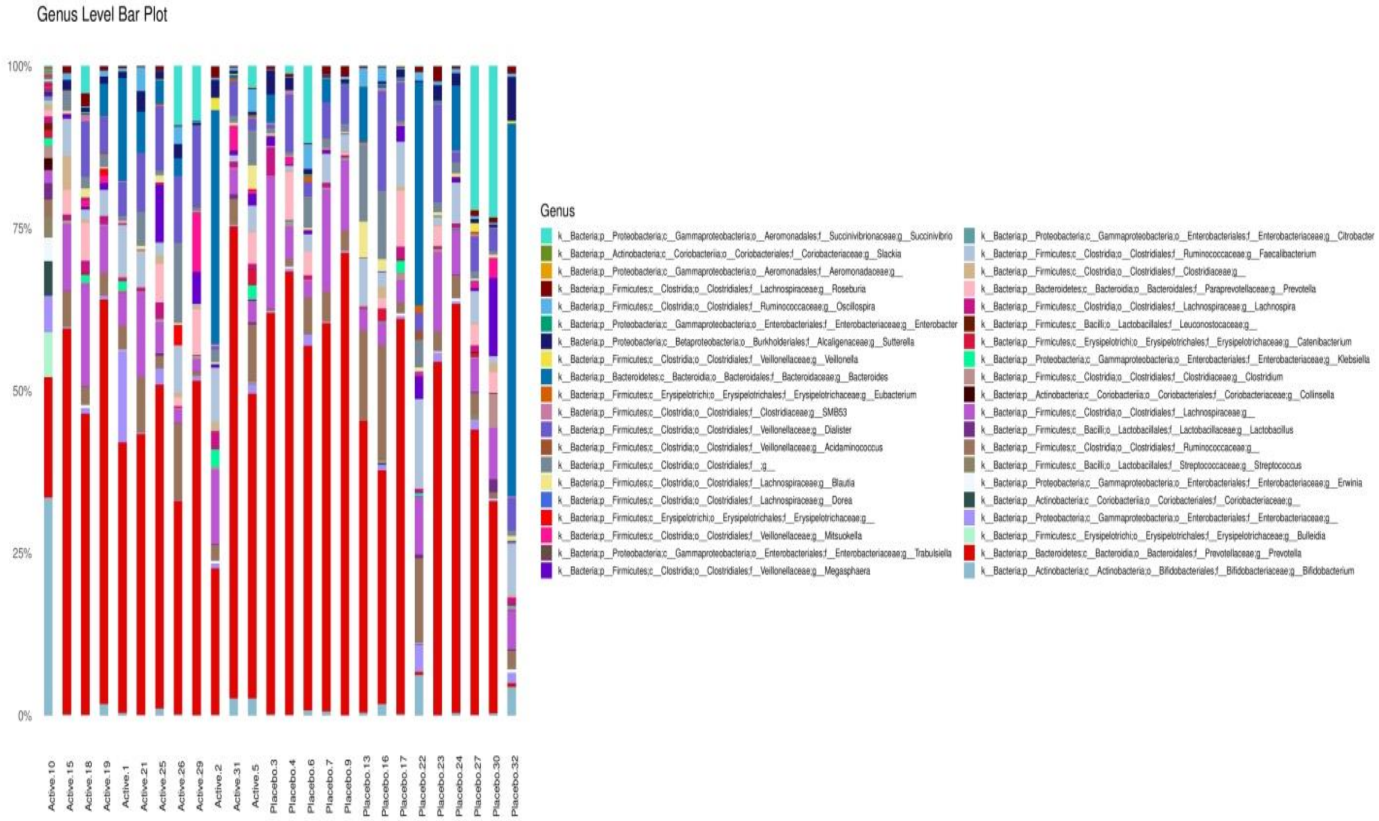

Figure S2: Microbial composition of active and placebo group at the genus level with corresponding relative percentages using the relative abundance (y-axis) values for all the samples(x-axis) with respect to their baseline and final visit.
